# Sulforaphane Effects on Neuronal-like Cells and Peripheral Blood Mononuclear Cells Exposed to 2.45 GHz Electromagnetic Radiation

**DOI:** 10.3390/ijms25147872

**Published:** 2024-07-18

**Authors:** Maria Paola Bertuccio, Caterina Saija, Giuseppe Acri, Riccardo Ientile, Daniela Caccamo, Monica Currò

**Affiliations:** Department of Biomedical and Dental Sciences and Morpho-Functional Imaging, University of Messina, 98125 Messina, Italy; caterina.saija@unime.it (C.S.); giuseppe.acri@unime.it (G.A.); riccardo.ientile@unime.it (R.I.); monica.curro@unime.it (M.C.)

**Keywords:** sulforaphane, hormetic effects, electromagnetic radiation, neuronal-like cells, peripheral blood mononuclear cells

## Abstract

Exposure to 2.45 GHz electromagnetic radiation (EMR) emitted from commonly used devices has been reported to induce oxidative stress in several experimental models. Our study aims to evaluate the efficacy of sulforaphane, a well-known natural product, in preventing radiation-induced toxic effects caused by a 24 h exposure of SH-SY5Y neuronal-like cells and peripheral blood mononuclear cells (PBMCs) to 2.45 GHz EMR. Cells were exposed to radiation for 24 h in the presence or absence of sulforaphane at different concentrations (5–10–25 µg/mL). Cell viability, mitochondrial activity alterations, the transcription and protein levels of redox markers, and apoptosis-related genes were investigated. Our data showed a reduction in cell viability of both neuronal-like cells and PBMCs caused by EMR exposure and a protective effect of 5 µg/mL sulforaphane. The lowest sulforaphane concentration decreased ROS production and increased the Mitochondrial Transmembrane Potential (Δ*ψm*) and the NAD+/NADH ratio, which were altered by radiation exposure. Sulforaphane at higher concentrations displayed harmful effects. The hormetic behavior of sulforaphane was also evident after evaluating the expression of genes coding for Nrf2, SOD2, and changes in apoptosis markers. Our study underlined the vulnerability of neuronal-like cells to mitochondrial dysfunction and oxidative stress and the possibility of mitigating these effects by supplementation with sulforaphane. To our knowledge, there are no previous studies about the effects of SFN on these cells when exposed to 2.45 GHz electromagnetic radiation.

## 1. Introduction

Scientific evidence about electromagnetic radiation’s impact on health suggests the involvement of the electromagnetic field (EMF) in several diseases, such as cancer, neurological disorders, and immunological alterations [1,2,3,4]. These effects have been associated with the increase in reactive oxygen species (ROS) and consequent oxidative stress [5,6]. Moreover, recent reviews on low- and high-frequency magnetic fields and oxidative stress indicate that the EMF may play a role in the onset of several neurodegenerative diseases and contribute to reducing fertility through oxidative stress induction and related antioxidant defense system alteration [7,8]. In fact, excessive amounts of ROS can interfere with numerous vital cellular processes and functions, altering biochemical and signaling processes or causing oxidative damage to nucleic acids and proteins that result in alterations in proliferation, cellular differentiation, inflammation, neuronal activity, reproduction, and behavior [7,9].

Our study had previously demonstrated that 2.45 GHz electromagnetic radiation (EMR) emitted from commonly used devices is able to induce both ROS production and mitochondrial imbalance in neuronal-like cells and peripheral blood mononuclear cells (PBMCs). Furthermore, differentiated SH-SY5Y cells were more prone to develop oxidative stress than PBMCs after exposure to EMR; in fact, neuronal-like cells react to the pro-oxidant insult with an early antioxidant defense response and activating pro-apoptotic events [10].

Recent studies have been conducted in brain cell lines after pulsed high-power microwave (HPM) 3.5 GHz exposure, which was able to induce apoptosis-related events such as enhanced ROS production and increased oxidative DNA damage at higher dosages [11,12]. Notably, the exposure of melanoma and normal human dermal fibroblast cells to HPM showed differential effects, with microwaves able to selectively stimulate the viability and proliferation of only melanoma cells [13], highlighting once again how different cell types can respond in different ways to radiation.

Furthermore, due to the presence of various types of radiation in working and domestic environments, the effects of exposure to biological systems vary greatly and today represent an important area of research that includes both EMR biological effects and safety levels [14].

Several studies have been carried out to evaluate the effectiveness of natural antioxidants extracted from plants in preventing the onset of pathologies linked to EMR exposure. For example, β-glucan has been shown to attenuate oxidative skin injury caused by EMR in rats [15], olive leaf extract ameliorates glucose metabolism disorder by reducing the oxidative stress induced by 2.45 GHz Wi-Fi signals in rat tissues [16], and the green tea derivative catechin, a polyphenol compound with free radical scavenging functions, seems to protect rats’ heart tissue from microwave-induced oxidative damage [17]. 

Another natural product with a well-known antioxidant effect is sulforaphane (SFN), an isothiocyanate compound abundant in Brassicaceae or Cruciferae family plants [18].

SFN is known to be able to fight different types of cancer due to its ability to inhibit tumor proliferation by triggering apoptosis. This natural compound improves glucose tolerance and reduces fat accumulation, thus showing anti-diabetic and anti-obesity effects. The antioxidant, anti-inflammatory, and anti-apoptotic properties of SFN are also considered to provide benefits against neurodegenerative diseases [19].

Other than displaying anti-cancer, anti-inflammatory, and antioxidant effects [20,21,22], SFN has also been shown to be protective against radiation-induced skin injury (RISI), probably because of its capacity to suppress oxidative stress by up-regulating the expression and activity of Nuclear factor erythroid 2-related factor 2 (Nrf2) [23]. Along with these beneficial features, SFN shows hormetic effects, since subtoxic doses can lead to cellular stress response, and toxic doses can induce apoptosis [24,25]. 

It is interesting to note that the activation of Nrf2 is involved, in addition to the above-mentioned antioxidant activity, in tumor progression and drug resistance in several types of cancers, such as papillary thyroid cancer and prostate cancer [26,27]. In fact, sustained Nrf2 activity in cancer leads to resistance to chemo- and radiotherapy, and it is linked to a poor prognosis, making necessary the development of approaches to restrict Nrf2 activity and downstream effects and thus representing a promising target for the treatment of tumors [28].

Regarding clinical trials on humans, the Food and Drug Administration imposed a limit of 200 µmol SFN, considering this natural product relatively safe when using low doses and not very harmful at higher doses [29]. 

The aim of this study was to evaluate the efficacy of sulforaphane (SFN) in preventing or worsening radiation-induced damage caused by a 24 h exposure of neuronal-like cells and PBMCs to 2.45 GHz EMR.

## 2. Results

The background electromagnetic measured value was <0.35 V/m both inside and outside the incubator and with the signal generator switched off. 

The measurement of the electromagnetic field inside the incubator with the signal generator turned on and the electric field probe positioned at 20 cm from the source element antenna (the electric field probe was placed in the same place as the sample) provided an electromagnetic field of 1.80 ± 0.05 V/m.

**Figure 1:**
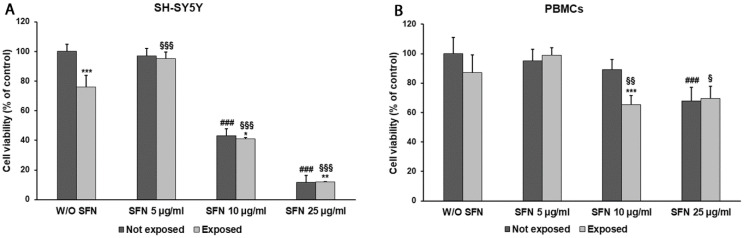
Cell viability in differentiated SH-SY5Y cells (**A**) and in PBMCs (**B**) not exposed (dark grey bars) and exposed (light grey bars) to 2.45 GHz radiation, without (W/O) SFN or with 5, 10, or 25 µg/mL of SFN. Values are means ± SD of five replicates. * *p* < 0.05, ** *p* < 0.01, and *** *p* < 0.001: significant differences between cells exposed and not exposed to 2.45 GHz radiation; ### *p* < 0.001: significant differences between cells not exposed to 2.45 GHz radiation and treated with 5, 10, or 25 µg/mL of SFN vs. cells not exposed to 2.45 GHz radiation W/O SFN; § *p* < 0.05, §§ *p* < 0.01, and §§§ *p* < 0.001: significant differences between cells exposed to 2.45 GHz radiation and treated with 5, 10, or 25 µg/mL of SFN vs. cells exposed to 2.45 GHz radiation W/O SFN.

After 24 h exposure to 2.45 GHz radiation, we observed a reduction in cell viability of both SH-SY5Y neuron-like cells and PBMCs (24% *p* = 0.0005 and 13% *p* = 0.1120, respectively), when compared to unexposed cells. The incubation with 5 µg/mL SFN showed a protective effect, increasing cell viability by 25% (*p* < 0.0001) in differentiated SH-SY5Y and by 14% (*p* = 0.1363) in PBMCs. At higher concentrations, SFN produced a toxic effect on SH-SY5Y cells, since it decreased cell viability in both radiation-exposed and unexposed cells. This effect was already evident in the presence of 10 µg/mL SFN, which induced a cell viability decrement of 57% (*p* < 0.0001) in unexposed cells and 46% (*p* < 0.0001) in radiation-exposed cells and to a greater extent in the presence of 25 µg/mL SFN (88% *p* < 0.0001 in unexposed, 84% *p* < 0.0001 in radiation-exposed cells) (Figure 1A). In PBMCs, SFN toxicity became evident at the highest concentration of 25 µg/mL, decreasing cell viability by 32% (*p* = 0.0002) in unexposed cells and by 20% (*p* = 0.0221) in radiation-exposed cells compared with cells cultured without SFN. Instead, in the presence of 10 µg/mL SFN, unexposed cells were more viable than radiation-exposed ones, the latter showing a 26% decrement in cell viability (*p* = 0.0031) (Figure 1B).

**Figure 2:**
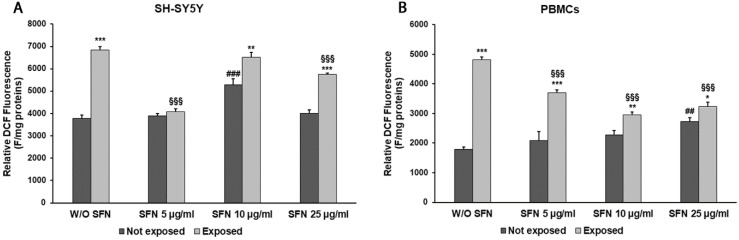
Changes in ROS production in differentiated SH-SY5Y cells (**A**) and in PBMCs (**B**) not exposed (dark grey bars) and exposed (light grey bars) to 2.45 GHz radiation, without (W/O) SFN or with 5, 10, or 25 µg/mL of SFN. Values are means ± SD of three replicates. * *p* < 0.05, ** *p* < 0.01, and *** *p* < 0.001: significant differences between cells exposed and not exposed to 2.45 GHz radiation; ## *p* < 0.01, and ### *p* < 0.001: significant differences between cells not exposed to 2.45 GHz radiation and treated with 5, 10, or 25 µg/mL of SFN vs. cells not exposed to 2.45 GHz radiation W/O SFN; §§§ *p* < 0.001: significant differences between cells exposed to 2.45 GHz radiation and treated with 5, 10, or 25 µg/mL of SFN vs. cells exposed to 2.45 GHz radiation W/O SFN.

As shown in Figure 2A,B, in the absence of SFN, an ROS production increment of 81% (*p* < 0.0001) was observed in cells exposed to 2.45 GHz radiation in differentiated SH-SY5Y cells and 170% (*p* < 0.0001) in PBMCs when compared with unexposed cells. This effect was reduced by the addition of the lowest SFN concentration, which decreased ROS production by 40% (*p* < 0.0001) and 23% (*p* < 0.0001), respectively, in radiation-exposed SH-SY5Y cells and PBMCs. Supporting the toxicity hypothesis of high SFN concentrations, an ROS production increment was observed also in unexposed SH-SY5Y neuronal-like cells in the presence of 10 µg/mL SFN (40%, *p* < 0.0001) (Figure 2A) and in unexposed PBMCs in the presence of 25 µg/mL SFN (53%, *p* = 0.0013) (Figure 2B). However, in PBMCs exposed to radiation, all SFN concentrations were proven to mitigate ROS production (Figure 2B). 

**Figure 3:**
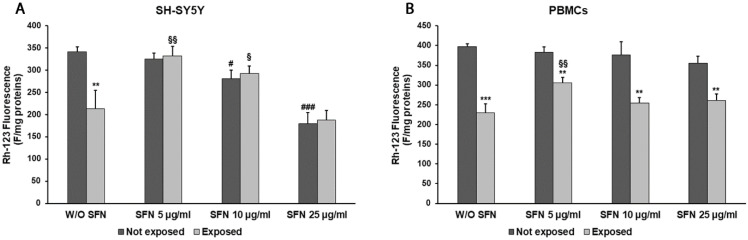
ΔΨm values in differentiated SH-SY5Y cells (**A**) and in PBMCs (**B**) not exposed (dark grey bars) and exposed (light grey bars) to 2.45 GHz radiation, without (W/O) SFN or with 5, 10, or 25 µg/mL of SFN. Values are means ± SD of three replicates. ** *p* < 0.01 and *** *p* < 0.001: significant differences between cells exposed and not exposed to 2.45 GHz radiation; # *p* < 0.05 and ### *p* < 0.001: significant differences between cells not exposed to 2.45 GHz radiation and treated with 5, 10, or 25 µg/mL of SFN vs. cells not exposed to 2.45 GHz radiation W/O SFN; § *p* < 0.05, §§ *p* < 0.01: significant differences between cells exposed to 2.45 GHz radiation and treated with 5, 10, or 25 µg/mL of SFN vs. cells exposed to 2.45 GHz radiation W/O SFN.

As expected, the exposure to 2.45 GHz radiation in the absence of SFN resulted in a ΔΨm decrement in both cell types (Figure 3). In SH-SY5Y cells, SFN, at doses of 5 and 10 µg/mL, induced an increment of ΔΨm (56%, *p* = 0.0029 and 38%, *p* = 0.0283, respectively) in cells exposed to radiation compared to cells exposed in the absence of SFN. Higher SFN concentrations (10 and 25 µg/mL) produced a statistically significant reduction in ΔΨm as well as in SH-SY5Y not exposed to radiation in comparison to control cells (18% *p* = 0.0146 and 48% *p* < 0.0001, respectively) (Figure 3A). 

In PBMCs, only the lowest SFN concentration (5 µg/mL) was able to partially reverse the radiation effects on ΔΨm. Indeed, the addition of 5 µg/mL SFN in radiation-exposed cells produced a 56% increment in ΔΨm (*p* = 0.0026) in comparison with radiation-exposed cells in the absence of SFN (Figure 3B), while other concentrations appeared ineffective. 

**Figure 4:**
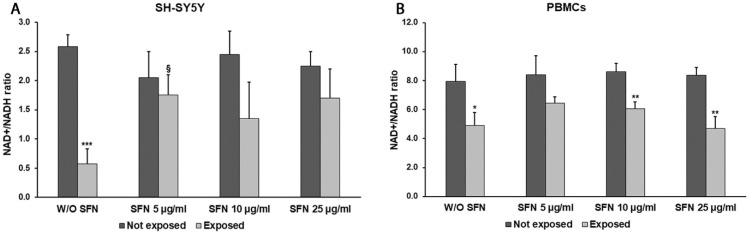
The NAD+/NADH ratio in differentiated SH-SY5Y cells (**A**) and in PBMCs (**B**) not exposed (dark grey bars) and exposed (light grey bars) to 2.45 GHz radiation without (W/O) SFN or with 5, 10, or 25 µg/mL of SFN. Values are means ± SD of three replicates. * *p* < 0.05, ** *p* < 0.01 and *** *p* < 0.001: significant differences between cells exposed and not exposed to 2.45 GHz radiation; § *p* < 0.05: significant difference between cells exposed to 2.45 GHz radiation and treated with 5, 10, or 25 µg/mL of SFN vs. cells exposed to 2.45 GHz radiation W/O SFN.

The effects of SFN on mitochondrial activity were also evaluated through the NAD+/NADH ratio measurement (Figure 4). In the absence of SFN, a significant decrement of the NAD+/NADH ratio was observed in radiation-exposed SH-SY5Y cells (78%, *p* = 0.0004) and PBMCs cells (53%, *p* = 0.0257) in comparison to unexposed cells. In general, the addition of SFN in cells exposed to radiation resulted in the NAD+/NADH ratio increasing at all SFN concentrations in comparison with cells exposed in the absence of SFN, even if statistically significant values were detected only at the lowest SFN concentration in SH-SY5Y cells (202%, *p* = 0.0478). 

**Figure 5:**
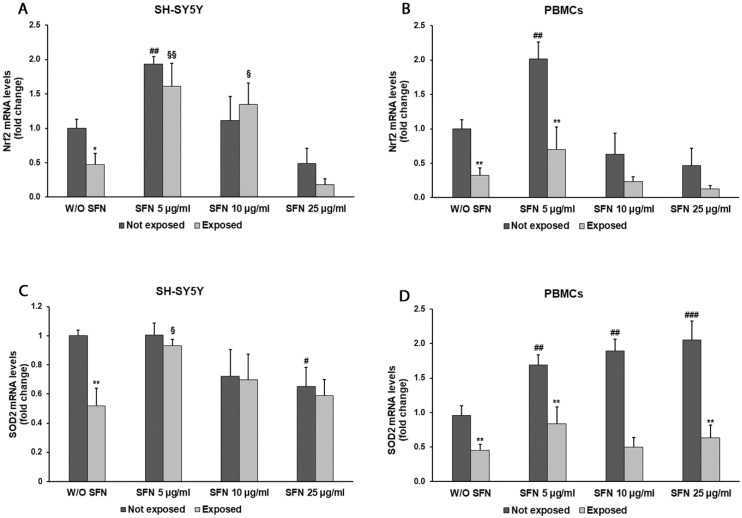
Nrf2 gene expression analyzed by real-time PCR in differentiated SH-SY5Y cells (**A**) and in PBMCs (**B**) and the SOD2 gene expression in differentiated SH-SY5Y cells (**C**) and in PBMCs (**D**) not exposed (dark grey bars) and exposed (light grey bars) to 2.45 GHz radiation without (W/O) SFN or with 5, 10, or 25 µg/mL of SFN. Values are means ± SD of three replicates. * *p* < 0.05, ** *p* < 0.01: significant differences between cells exposed and not exposed to 2.45 GHz radiation; # *p* < 0.05, ## *p* < 0.01 and ### *p* < 0.001: significant differences between cells not exposed to 2.45 GHz radiation and treated with 5, 10, or 25 µg/mL of SFN vs. cells not exposed to 2.45 GHz radiation W/O SFN; § *p* < 0.05, §§ *p* < 0.01: significant difference between cells exposed to 2.45 GHz radiation and treated with 5, 10, or 25 µg/mL of SFN vs. cells exposed to 2.45 GHz radiation W/O SFN.

In order to evaluate the effects of SFN on redox markers in cells exposed to radiation, we measured the expression of Nrf2 and SOD2 genes, coding for antioxidant response-related proteins (Figure 5). Radiation exposure caused a reduction in Nrf2 expression in SH-SY5Y and PBMCs cultured in the absence of SFN (Figure 5A,B). The addition of 5 µg/mL SFN led to a 3.4-fold increase in Nrf2 expression (*p* = 0.0022) in SH-SY5Y-exposed cells and a 1.9-fold increase (*p* = 0.0043) in unexposed cells in comparison with cells cultured in the absence of SFN. A 2.4-fold increase (*p* = 0.0104) was also seen with the addition of 10 µg/mL SFN in radiation-exposed cells in comparison with unexposed ones, while the highest SFN concentration caused a decrease in Nrf2 expression in both exposed and unexposed cells in comparison with cells cultured in the absence of SFN (Figure 5A). In PBMCs, SFN was effective in promoting Nrf2 expression only at the lowest concentration used; in fact, gene expression increased by 2.3 times (*p* = 0.0968) in radiation-exposed cells and by 2 times (*p* = 0.0048) in unexposed cells in comparison with cells cultured in the absence of SFN (Figure 5B).

While evaluating SOD2 expression in SH-SY5Y cells, we observed a 47% decrement (*p* = 0.0015) in cells exposed to radiation compared to unexposed ones without the addition of SFN, while in cells cultured with SFN, SOD2 expression was similar in cells exposed and not exposed to radiation. A 76% increase in SOD2 expression (*p* = 0.0113) was observed in cells exposed to radiation in the presence of 5 µg/mL SFN when compared to exposed cells not treated with the natural compound (Figure 5C). 

Moreover, our data showed that 5, 10, and 25 µg/mL SFN increased by 1.7 times (*p* = 0.0094), 1.9 times (*p* = 0.0020), and 2.1 times (*p* = 0.0005) mitochondrial SOD transcript levels in PBMCs not exposed to radiation. On the contrary, no significant differences were detected between cells exposed to radiation in the absence or presence of SFN at any concentration (Figure 5D).

**Figure 6:**
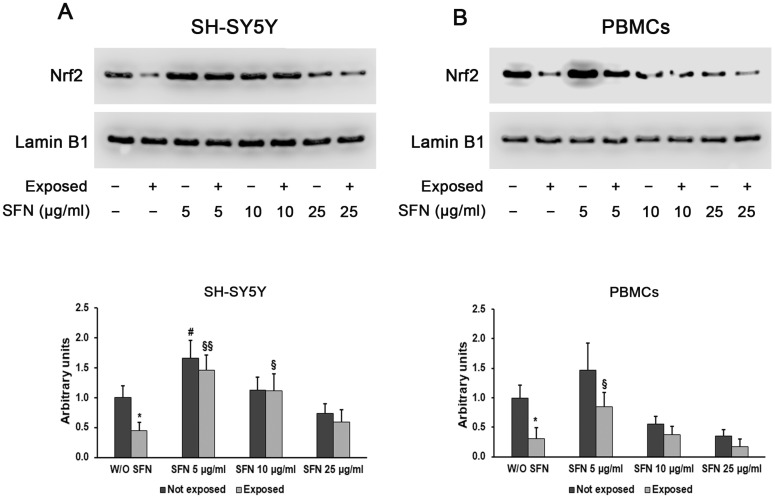
Evaluation of Nrf2 protein expression by Western blotting in differentiated SH-SY5Y cells (**A**) and PBMCs (**B**), not exposed (dark grey bars) and exposed (light grey bars) to 2.45 GHz radiation, without (W/O) SFN or with 5, 10, or 25 µg/mL of SFN. Densitometric analysis of Nrf2 was carried out after normalization against lamin B1. Values are means ± SD of three replicates. * *p* < 0.05: significant differences between cells exposed and not exposed to 2.45 GHz radiation; # *p* < 0.05: significant differences between cells not exposed to 2.45 GHz radiation and treated with 5, 10, or 25 µg/mL of SFN vs. cells not exposed to 2.45 GHz radiation W/O SFN; § *p* < 0.05, §§ *p* < 0.01: significant differences between cells exposed to 2.45 GHz radiation and treated with 5, 10, or 25 µg/mL of SFN vs. cells exposed to 2.45 GHz radiation W/O SFN.

Western blot analysis confirmed the real-time PCR results showing a reduction in the Nrf2 protein level in SH-SY5Y and PBMCs cultured without SFN after radiation exposure and an increase in Nrf2 levels after the addition of 5 µg/mL of SFN. A decrease in protein content is visible using higher SFN concentrations (Figure 6).

To investigate the effect of SFN on apoptosis generated by radiation exposure, we measured the BAX/BCL2 ratio, the first one being a pro-apoptotic and the second one an anti-apoptotic marker.

**Figure 7:**
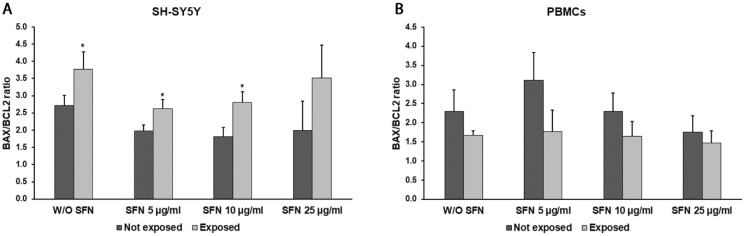
BAX/BCL2 ratio analyzed by real-time PCR in differentiated SH-SY5Y cells (**A**) and in PBMCs (**B**) not exposed (dark grey bars) and exposed (light grey bars) to 2.45 GHz radiation without (W/O) SFN or with 5, 10, or 25 µg/mL of SFN. Values are means ± SD of three replicates. * *p* < 0.05: significant differences between cells exposed and not exposed to 2.45 GHz radiation.

As shown in Figure 7, the results obtained in the two cell types are quite different. In fact, the exposure to 2.45 GHz radiation in the presence or absence of SFN caused an increment of the BAX/BCL2 ratio in SH-SY5Y cells compared with unexposed cells. The levels of the BAX/BCL2 ratio decreased in a non-significant manner in radiation-exposed cells in the presence of either 5 µg/mL SFN or 10 µg/mL SFN compared with radiation-exposed cells in the absence of SFN (Figure 7A).

With regard to the SFN effects in PBMCs, we could notice that there were no significant differences in the BAX/BCL2 ratio between radiation-exposed cells and unexposed ones, in the presence or absence of SFN (Figure 7B).

To better define the involvement of the apoptotic pathway in response to 2.45 GHz exposure and SFN treatments, we evaluated the caspase-3 activity (Table 1). In accordance with the results obtained by BAX/BCL2 measurements, the caspase-3 activity increased in SH-SY5Y cells after exposure to 2.45 GHz radiation when compared with cells not exposed, both in the absence of SFN (*p* = 0.0160) and with 10 µg/mL of SFN (*p* = 0.0267). In unexposed cells, the addition of 10 µg/mL of SFN resulted in a significative reduction in the caspase-3 activity in comparison with cells cultured without SFN (*p* = 0.0348). The activity of caspase-3 decreased also in cells exposed to radiation and cultured with 5 and 10 µg/mL of SFN when compared to exposed cells in the absence of SFN (*p* = 0.0117 and *p* = 0.0194, respectively).

Moreover, according to BAX/BCL2 ratio data, SFN did not significantly change the caspase-3 activity in PBMCs exposed and not exposed to radiation.

## 3. Discussion

In this work, we evaluated the protective/toxic effect of the natural product sulforaphane on cells “stressed” by 2.45 GHz EMR, whose harmful effects are well known. To our knowledge, there are no previous studies about the effects of SFN on these cells exposed to 2.45 GHz electromagnetic radiation. Our findings are consistent with our previous observations, demonstrating that exposure to EMR decreases cell viability in comparison to unexposed cells [10], and with several reports showing a dose-dependent viability reduction in cells cultured with SFN [18,30,31]. Furthermore, 5 µg/mL SFN showed a protective effect by preventing cell death and oxidative stress development caused by exposure to radiation in both SH-SY5Y neuronal-like cells and PBMCs.

As reported by Stefi and colleagues, SH-SY5Y exposure to EMF increases ROS levels and mitochondrial damage [32]. Similar results were obtained by Kazemi and colleagues (2015) in monocyte-rich PBMCs when exposed to 900 MHz EMF [33]. Ran Y. et al. (2023) have already demonstrated the SFN capacity to reduce the level of oxidative stress induced by ionizing radiation [34]. Here, we showed that, at a low concentration, SFN was able to reduce ROS production and restore mitochondrial permeability following damage to cells exposed to EMR. On the contrary, high SFN concentrations were not able to counteract the negative effects of radiation and, in turn, were harmful by increasing ROS production and modifying mitochondrial permeability. The biological activity of SFN changes depending on the dose used, and this ability is called hormesis; usually, this natural compound acts as an indirect antioxidant, and subtoxic doses induce an adaptive cellular response to stress, while using toxic doses can lead to cell death [18]. The SFN-mediated increment of ROS production has been associated with apoptotic pathway activation in various cancer types, i.e., bladder cancer, hepatocellular carcinoma, lung cancer, and breast cancer [35,36,37,38], showing a central role in preventing cancer progression. Using PBMCs, Bessler and Djaldetti (2018) found that SFN addition was able to modify cell-mediated immune responses, affecting tumorigenesis and metastasis [39]. Our findings suggest that PBMCs appear more resistant to oxidative stress caused by high SFN concentrations than differentiated SH-SY5Y cells. 

Changes in oxidative metabolism can also be assessed by evaluating variations in the NAD+/NADH ratio. In fact, preserving this ratio is crucial to mitochondrial function, the as NAD+/NADH pair links substrate oxidation by the tricarboxylic acid (TCA) cycle to the creation of adenosine triphosphate (ATP) carried out by the electron transport chain (ETC) and oxidative phosphorylation [40].

Kalpana Deepa Priya and colleagues (2011) proved that SFN (9 µmol/mice/day) can preserve mitochondria functionality. In fact, in their study, the inactivated TCA cycle enzymes in animals treated with the carcinogen benzo(a)pyrene recovered almost fully upon the increase in antioxidant enzyme activity in SFN-treated groups [41]. Here, we showed that SFN was able to partially counteract the decrease in the NAD+/NADH ratio caused by cell exposure to 2.45 GHz radiation, and this ability was evident, especially in neuronal-like cells.

Cell redox balance is maintained also thanks to the NRF2 capacity to offset mitochondrial ROS production [42,43]. Our data showed that cell exposure to EMR down-regulated Nrf2 expression likely through the activation of kinases. In fact, glycogen synthase kinase-3β is able to induce Nrf2 degradation by the proteasome [44], and Serin M and colleagues already demonstrated an overexpression of this gene in cells exposed to 2.4–3 GHz Wi-Fi RF-EMF [45]. Moreover, several studies have shown that SFN is a potent activator of the transcription factor Nrf2 [21,46,47,48]. This activation results in blocking oxidative stress through the increase in the expression of Nrf2-targeted antioxidant genes, such as glutathione peroxidase (GPx), superoxide dismutase (SOD), and catalase (CAT) [49]. Our data confirmed these assumptions, since SOD2 expression decreased in both neuronal-like cells and PBMCs after exposure to radiation, while after SFN addition, the expression of Nrf2 and SOD2 increased. However, it should be noted that the use of high SFN concentrations did not achieve the same results, since the expression of both Nrf2 and SOD2 decreased after the addition of 25 µg/mL SFN, probably due to the hormetic properties of this natural product.

As previously reported, exposure to 2.45 GHz EMR led to an increase in apoptosis in neuronal-like cells but not in PBMCs [10]. SFN treatment has been associated with BAX/BCL2 ratio alteration [38], and Schepici G. et al. (2020) reported that pretreatment with SFN (1 µM, 2 µM, and 5 µM) protected the cells from apoptosis in a dose-dependent way. It seems that SFN reduced apoptosis by up-regulating BCL-2 expression and reducing the activation of the proapoptotic protein BAX [50]. The antiapoptotic effect of SFN is also demonstrated by its ability to decrease caspase-3 activity [51]. On the contrary, thanks to its anti-cancer properties, SFN is able to induce apoptosis at higher doses as reported by Hussain and colleagues, who found an apoptosis induction in human breast cells through BCL-2 down-regulation after the treatment with 25 µM SFN [52]. We found a not significant reduction in the BAX/BCL2 ratio after SH-SY5Y cells were treated with 5 and 10 µg/mL SFN. On the other hand, we have once again demonstrated the lack of induction of apoptosis following a 24 h exposure of PBMCs to EMR, an induction which is also absent following treatment with different SFN concentrations. 

Further in vivo studies could be carried out on individuals with electromagnetic hypersensitivity, to evaluate the efficacy of sulforaphane on EMR exposure’s harmful effects in these subjects. 

## 4. Materials and Methods

### 4.1. Cell Culture Conditions 

The SH-SY5Y cell line (CRL-2266) was obtained from the American Type Culture Collections (ATCC) (Rockville, MD, USA). These cells were cultured in equal amounts of minimum essential medium (MEM) eagle (M5650) and Nutrient Mixture F-12 Ham (M4888) (Sigma, Milan, Italy) with additional 10% (*v*/*v*) heat-inactivated fetal bovine serum (FBS) (35-079-CV, Corning, Woodland, CA, USA), 2 mM L-glutamine (ECB3000D, EuroClone, Milan, Italy), 1 mM sodium pyruvate (25-000-CI, Corning, Manassas, VA, USA), and 1% of antibiotic mixture of 10 U/mL penicillin and 100 mg/mL streptomycin (ECB3001D, EuroClone, Milan, Italy). A total of 5 × 10^5^ cells/mL were seeded in 6-well plates and maintained at 37 °C in a humidified 5% CO_2_ atmosphere.

SH-SY5Y cells were differentiated into neuronal-like cells as previously described by Condello S. et al. 2012 [53]. Briefly, a mixture of MEM/Ham’s F12 medium containing 10 µM retinoic acid (RA) (R2625, Sigma-Aldrich, Milan, Italy) 10 mM in dimethyl sulfoxide (DMSO) stock solution, 1% FBS, 2 mM L-glutamine, and 1 mM sodium pyruvate was added to sub-confluent cells and renewed every 2 days.

After 5 days of cells exposure to 10 µM RA, SFN with a concentration of 5 µg/mL, 10 µg/mL, or 25 µg/mL (starting solution of 5 mg/mL in DMSO) in the form of R-sulforaphane (≥95% -HPLC) (S6317, Sigma-Aldrich, Darmstadt, Germany) in medium with 2% FBS was added to the 6-well plates, and cells were exposed to 2.45 GHz for 24 h at 37 °C in a humidified 5% CO_2_ atmosphere. 

### 4.2. PBMC Culture Conditions

PBMC separations were performed from 3 blood transfusion bags (150 mL) collected from healthy volunteers using Histopaque^®^-1077 (10771, Sigma, Milan, Italy) following the manufacturer’s instructions. After two consecutive washes in phosphate-buffered saline (PBS) solution (P4474, Sigma, Milan, Italy), the PBMC suspension was collected by centrifugation and then cultured at a density of 4 × 10^6^ cells/well in 6-well plates with RPMI-1640 medium (15-041-CVR, Sigma, Milan, Italy), with the addition of 10% FBS and 1% standard antibiotics mixture. Cells were incubated with three different concentrations of SFN (5 µg/mL, 10 µg/mL, and 25 µg/mL) at 37 °C in a humidified 5% CO_2_ atmosphere and exposed to 2.45 GHz EMR for 24 h.

Volunteers were all white/Caucasian males with a mean age of 35 (±5) and a body mass index (BMI) between 18.5 and 24.9 (kg/m^2^).

### 4.3. Experimental Design 

As already described in our previous work [10], the experimental setup used to generate EMR was represented by a biconic transmitting antenna (RKB), working in the frequency range of 1.7–2.5 GHz, with an isotropic gain of about 0 dBmW in the same range, connected by a cable to a signal generator Hewlett–Packard 8648C (Agilent Technologies, Santa Clara, CA, USA), working in the frequency range 100 kHz–3200 MHz (output RF level ranging from −136 dBmW to +20 dBmW). 

The output power of 0 dB (mW) corresponding to 1 mW and a frequency of 2.45 GHz was set.

The antenna was located in an incubator (series 5400-115V models, Thermo Electron Corporation, Winchester) in 5% CO_2_ and 95% air humidified at the temperature of 37 °C.

Continuous microwave radiation was used to irradiate the samples. The electromagnetic field exposures were evaluated by using the PMM 5083-b (Narda Safety Test Solutions, Cisano sul Neva (Savona)—Italy) portable field meter, coupled with a triaxial electric field probe EP745 (frequency range 100–7 GHz and dynamic range 0.35–450 V/m). The electromagnetic field measurements were conducted outside and inside the incubator with the signal generator switched off and switched on.

The 6-well plates of differentiated SH-SY5Y cells or lymphocytes treated or not with SFN were exposed to electromagnetic fields for 24 h. The plates were placed approximately 10 cm from the antenna at the center of a uniform field area to avoid the heat produced by the device. 

Control cells not exposed to EMR were placed into another incubator of the same model in 5% CO_2_ and 95% air humidified at the temperature of 37 °C. 

### 4.4. Cell Viability Assay

To evaluate cell viability, we performed a quantitative colorimetric assay with 3-[4,5-dimethylthiazol-2-yl]-2,5-diphenyltetrazolium bromide (MTT) (CT01-5, Merck Millipore, Billerica, MA, USA). SH-SY5Y cells and PBMCs were cultured in 96-well culture plates at a density of 5 × 10^4^ and 1 × 10^4^ cells/well, respectively, and incubated with fresh medium containing MTT (0.5 mg/mL) at 37 °C for 4 h. Before the SFN addition and EMR exposure, SH-SY5Y cells were differentiated with RA as previously reported.

Insoluble formazan crystals were dissolved in 100 µL of a 0.04 N HCl/isopropanol solution at 37 °C for 1 h. The formazan absorbance at 570 nm, in each well, was evaluated using a microplate reader (Tecan Italia, Milan, Italy). 

Analysis was performed in quintuplicate.

### 4.5. Intracellular ROS Assessment

To evaluate the intracellular ROS content, we used the non-fluorescent probe 2′,7′-dichlorofluorescein diacetate (H2DCF-DA) (D6883, Sigma, Milan, Italy) that, reacting with ROS, is able to generate a fluorescent product. After 24 h of treatment, 5 µM of H2DCF-DA dissolved in DMSO was added in the medium of differentiated SH-SY5Y and left for 30 min at 37 °C in the dark. SH-SY5Y cells were then washed two times with PBS (pH 7.4), collected with non-enzymatic cell dissociation solution, resuspended in PBS (with the addition of 0.1 M KH_2_PO_4_ and 0.5% Triton X-100), and centrifugated at 13,000 rpm for 10 min. Finally, the fluorescence intensity was evaluated in the supernatants at an excitation wavelength of 480 nm and an emission wavelength of 540 nm by a microplate reader (Tecan Italia, Milan, Italy). 

Similarly, PBMCs were incubated with 5 µM H2DCF-DA for 30 min at 37 °C, and after two washes with PBS (pH 7.4), the cells were resuspended in PBS with the supplementation of 0.1 M KH_2_PO_4_ and 0.5% Triton X-100. After centrifugation, the fluorescence intensity was evaluated in the supernatants as previously described. 

The protein content of the cells’ lysates was established using the Lowry method, and these values were used to normalize the DCF fluorescence. Each assay was performed in triplicate.

### 4.6. Mitochondrial Transmembrane Potential (Δψm) Assessment

The cationic fluorescent dye Rhodamine 123 (Rh-123) (D23806, Thermo Fisher Scientific, Eugene, OR, USA) was used to evaluate cellular Δ*ψ*m. SH-SY5Y and PBMCs, after treatments previously described, were incubated with 10 µM Rh-123 added in the fresh medium, for 30 min at 37 °C in the dark. After two washes with PBS (pH 7.4), the fluorescence intensity was evaluated using a wavelength of 488 nm excitation and 525 nm emission in a microplate reader (Tecan Italia, Milan, Italy). The Lowry method was applied to assess the protein content in cell lysates; thus, the Rh-123 fluorescence was normalized for the total protein amount. Each assay was performed in triplicate.

### 4.7. NAD+/NADH Ratio 

The colorimetric NAD/NADH Assay AmpliteTM Kit (15273, AAT BioQuest, Sunnyvale, CA, USA) was used to evaluate the NAD+/NADH ratio amount in neuronal-like cells and PBMCs according to the manufacturer’s instructions. 

The absorbance was read on a microplate reader at 450 nm (Tecan Italia, Milan, Italy). Each assay was performed in triplicate.

### 4.8. Real-Time PCR Analysis

RNA was extracted from cells using Trifast reagent (EMR517100, Euroclone, Milan, Italy). A High-Capacity cDNA Archive kit (4368814, Thermo Fisher Scientific, Rockford, IL, USA) was used to reverse transcribe two micrograms of RNA according to the manufacturer’s instructions. mRNA levels of Nrf2, SOD2, BAX, BCL2, and β-Actin (endogenous control) were detected by Real-time PCR using SYBR green-based gene expression analysis. The forward and reverse sequences of the primers used in our experiments (Metabion International AG, Planegg, Germany) are reported in Table 2.

Real-time PCR experiments were carried out in triplicate in a 96-well plate using a reaction mix containing 0.1 μM specific primers, 25 ng cDNA, and SYBR green PCR Master mix (1X) (4472908, Thermo Fisher Scientific, Vilnius, Lithuania), in a final volume of 10 μL. A 7900HT Fast Real-Time PCR System (Applied Biosystems, Foster City, CA, USA) was used to perform the PCR reactions with the following steps: 1 cycle at 95 °C for 10 min, followed by 45 cycles at 95 °C for 15 s and 60 °C for 1 min for all the genes, except for Nrf2, for which the following profile was performed: 1 cycle at 95 °C for 10 min, followed by 45 cycles at 95 °C for 15 s and 55 °C for 1 min. Primer annealing specificity was tested by adding a standard dissociation stage.

### 4.9. Western Blotting

Western blot analysis was performed to detect the presence of Nrf2 in the nuclear compartment of SH-SY5Y cells and PBMCs. nuclear extracts from exposed and unexposed cells were prepared as previously described [54]. Then, 20 µg of protein was loaded on a 10% denaturing SDS-polyacrylamide gel and transferred to nitrocellulose membranes. Membranes were probed with primary antibodies against Nrf2 (AB31163, Abcam, Italy) and lamin B1 (SAB1306342, Sigma-Aldrich, Darmstadt, Germany), both diluted 1:1000, followed by incubation with horseradish-peroxidase-conjugated anti-rabbit secondary antibody (diluted 1:5000) (A9169, Sigma-Aldrich, Darmstadt, Germany).

Immunoblots were developed using an ECL chemiluminescent detection system kit (34580, Thermo Fisher Scientific, Rockford, IL, USA), and the signal was detected and quantified by scanning densitometry with a bio-image analysis system (C-DiGit, Li-cor, Lincoln, NE, USA).

### 4.10. Caspase-3 Activity

Caspase-3 activity was measured with a colorimetric kit (CASP3C, Sigma-Aldrich, Saint Louis, MO, USA) using the DEVD-pNa substrate, according to the manufacturer’s instructions. Briefly, aliquots from cell lysates were incubated at 37 °C overnight, and then the DEVD-pNa cleavage was evaluated by measuring the sample absorbance at 405 nm, using a DU 800 spectrophotometer (Beckman Coulter, Fullerton, CA, USA).

Measurements were normalized by protein content and calibrated against a standard linear regression curve of p-Na. Caspase activity was defined as nmol pNA released per hour per mg of protein (nmol/h/mg protein) [55]. All the experiments were set up in triplicate in a 96-well plate.

### 4.11. Statistical Analysis

Obtained data are expressed as mean ± standard error of the mean (SEM). Student’s *t*-test for comparisons between two groups was used to perform statistical analysis. *p* values less than 0.05 were considered significant.

## 5. Conclusions

Our findings underlined the vulnerability of neuronal-like cells to mitochondrial dysfunction and oxidative stress, which is probably because of the high energy requirements of the central nervous system. Furthermore, we highlighted the antioxidant properties of SFN in this cell type. In fact, this biological compound was able to reduce the damage induced by exposure to 2.45 GHz EMR by protecting cells from oxidative stress and mitochondrial alterations. At the same time, the hormetic “character” of SFN was evident. In fact, this natural compound was harmful at higher concentrations both in neuronal-like cells and in PBMCs, underlying the necessity to pay attention to incorrectly dosed dietary supplements.

## Figures and Tables

**Table 1:** Caspase-3 activity (pmol DEVD-pNA/h/mg prot) in differentiated SH-SY5Y cells and PBMCs, exposed and not exposed to 2.45 GHz radiation, cultured with or without (W/O) SFN.

SFN	2.45 GHz Radiation Exposure	SH-SY5Y	PBMCs
W/O	-	5.06 ± 0.7	1.90 ± 0.5
	+	7.52 ± 0.8 *	1.56 ± 0.3
5 µg/mL	-	3.74 ± 0.9	2.57 ± 0.4
	+	4.98 ± 0.6 §	1.61 ± 0.5
10 µg/mL	-	3.50 ± 0.5 #	1.69 ± 0.5
	+	5.20 ± 0.7 *, §	1.55 ± 0.4
25 µg/mL	-	4.72 ± 1.0	1.45 ± 0.4
	+	5.81 ± 1.5	1.37 ± 0.3

Values are means ± SD of three replicates. * *p* < 0.05: significant differences between cells exposed and not exposed to 2.45 GHz radiation; # *p* < 0.05: significant differences between cells not exposed to 2.45 GHz radiation and treated with 5, 10, or 25 µg/mL of SFN vs. cells not exposed to 2.45 GHz radiation W/O SFN; § *p* < 0.05: significant differences between cells exposed to 2.45 GHz radiation and treated with 5, 10, or 25 µg/mL of SFN vs. cells exposed to 2.45 GHz radiation W/O SFN.

**Table 2:** Primer sequences used for real-time PCR experiments.

Target	Primer Sequence 5′ > 3′	
	Forward	Reverse
*β-actin*	TTGTTACAGGAAGTCCCTTGCC	ATGCTATCACCTCCCCTGTGTG
*Nrf2*	CATCACCAGAACACTCAG	CTTCCACTTCAGAATCACT
*SOD2*	TGCTGCTTGTCCAAATCAGG	CACACATCAATCCCCAGCAGT
*BAX*	GGACGAACTGGACAGTAACATGG	GCAAAGTAGAAAAGGGCGACAAC
*BCL2*	ATCGCCCTGTGGATGACTGAG	CAGCCAGGAGAAATCAAACAGAGG

## Data Availability

The raw data supporting the conclusions of this article will be made available by the authors, upon reasonable request.
